# ﻿Identification and phylogenetic analysis in *Pterorhinuschinensis* (Aves, Passeriformes, Leiothrichidae) based on complete mitogenome

**DOI:** 10.3897/zookeys.1172.107098

**Published:** 2023-07-21

**Authors:** Guirong Bai, Qingmiao Yuan, Qiang Guo, Yubao Duan

**Affiliations:** 1 Key Laboratory for Conserving Wildlife with Small Populations in Yunnan, Southwest Forestry University, Kunming 650224, China Southwest Forestry University Kunming China; 2 Department of Biodiversity Conservation, Southwest Forestry University, Kunming 650224, China Southwest Forestry University Kunming China

**Keywords:** Black-throated Laughingthrush, duplicate control region, mitogenome, phylogeny, reclassification, taxonomy

## Abstract

The Black-throated Laughingthrush (*Pterorhinuschinensis*) is a bird belonging to the order Passeriformes and the family Leiothrichidae, and is found in Cambodia, China, Laos, Myanmar, Thailand and Vietnam. *Pterorhinuschinensis* was once classified as belonging to the genus *Garrulax*. However, recent research has reclassified it in the genus *Pterorhinus*. In this study, we sequenced and characterized the complete mitogenome of *P.chinensis*. The complete mitochondrial genome of *P.chinensis* is 17,827 bp in length. It consists of 13 PCGs, 22 tRNAs, two rRNAs, and two control regions. All genes are coded on the H-strand, except for one PCG (nad6) and eight tRNAs. All PCGs are initiated with ATG and stopped by five types of stop codons. Our comparative analyses show irregular gene rearrangement between trnT and trnP genes with another similar control region emerging between trnE and trnF genes compared with the ancestral mitochondrial gene order, called “duplicate CR gene order”. The phylogenetic position of *P.chinensis* and phylogenetic relationships among members of Leiothrichidae are assessed based on complete mitogenomes. Phylogenetic relationships based on Bayesian inference and maximum likelihood methods showed that *Garrulax* and (*Pterorhinus* + *Ianthocincla*) formed a clade. *Leiothrix* and *Liocichla* also formed a clade. Our study provides support for the transfer of *P.chinensis* from *Garrulax* to *Pterorhinus*. Our results provide mitochondrial genome data to further understand the mitochondrial genome characteristics and taxonomic status of Leiothrichidae.

## ﻿Introduction

The mitochondrial genome (hereafter mitogenome) has proven very useful in phylogenetics and population genetics of avian taxa (Mindell et al. 1998; Jønsson et al. 2019). Inherited exclusively from the mother, the mitochondrial genome is highly conserved, with little to no recombination within the mitogenome (Koehn and Nei 1983; Lansman et al. 1983). Moreover, mitochondrial genomes are comparatively more conserved than nuclear genomes during transition events in the context of evolution, especially in birds (Rheindt and Edwards 2011). Such unique architecture, in terms of organization as well as evolutionary behavior, enables mitochondrial genomes to carry phylogenetic information more consistently than nuclear markers (Rheindt and Edwards 2011). Birds, however, are particularly noteworthy because their mitochondrial genomes are characterized by a gene order different from that found in the majority of vertebrates due to rearrangements near the control region (Desjardins and Morais 1990). Most of the mitochondrial DNA mutations are point mutations, with few insertions or deletions. Moreover, as mitochondrial gene evolution rates differ (Aquadro and Greenberg 1983; Cann et al. 1984), different genes in the mitochondrial genome can be used to address different issues in phylogenetics and population genetics (Wenink et al. 1994). Mitochondrial genes are more closely linked and easier to identify than nuclear genes. Because of these advantages, the mitogenome has been widely used in studies of vertebrate phylogeny. Accordingly, an increasing number of complete sequences of mitogenomes from birds has been determined, and their structural features have been studied (Haddrath and Baker 2001; Krzeminska et al. 2016; Master et al. 2016).

The babblers are a diverse group of oscine passerine birds. Fregin et al. determined five primary clades at the rank of family for the babbler assemblage: Sylviidae, Zosteropidae, Timaliidae, Pellorneidae and Leiothrichidae (Fregin et al. 2012). This taxonomy has been gradually recognized in recent taxonomic lists (Dickinson and Christidis 2014). However, some phylogenetic relationships and genus names remain controversial. Leiothrichidae represents the largest clade of babblers in terms of species diversity (Cibois et al. 2018). Distributed throughout Africa, most of southern Asia, and the Great Sunda region, Leiothrichidae are most diverse in the Sino-Himalayan and South-East Asian regions. Leiothrichidae are generally rather large birds which inhabit the understory of thickets in mountainous areas, with many species foraging mainly on the ground in a thrush-like manner (Cibois et al. 2018).

The Black-throated Laughingthrush (*Pterorhinuschinensis* Scopoli, 1786) belongs to the order Passeriformes and the family Leiothrichidae. It is distributed in East Asia (China) and South and Southeast Asia (Cambodia, Laos, Myanmar, Thailand and Vietnam), and inhabits forest, shrubland and grassland (HBW-BirdLife 2022; IUCN 2023). This species is listed as Least Concern (LC) on the IUCN Red List, but the global population is decreasing (IUCN 2023). At present, the genus allocation of *P.chinensis* is controversial. It was once classified as *Garrulax* (Moyle et al. 2012). However, in a recent study, Cibois et al. (2018) collected the data of 102 species from 21 genera of Leiothrichidae, performed phylogenetic analysis and divergence time estimation based on molecular markers, and reclassified generic limits in combination with morphological characteristics. The results supported the transfer of 16 species from the original genus *Garrulax* to *Pterorhinus*, including the *P.chinensis*. Meanwhile, del Hoyo et al. (2016) and Cai et al. (2019) also support the classification of the Black-throated Laughingthrush into the genus *Pterorhinus*. In this study, we describe the complete mitogenome of *P.chinensis* and compare it with those of other species of Leiothrichidae. Our analysis of phylogenetic relationships confirms that *P.chinensis* should be classified as a member of *Pterorhinus*.

## ﻿Material and methods

### ﻿Samples and DNA extraction

The *P.chinensis* sample was collected in Dehong Dai and Jingpo Autonomous Prefecture (Yunnan, China) by the Dehong Prefecture Wildlife Shelter and Rescue Center and subsequently provided to the Department of Biodiversity Conservation, Southwest Forestry University. The tissue used in this study was preserved in absolute ethanol and stored at -20 °C until DNA extraction. Total DNA was extracted from muscle tissue of the bird using the TIANamp Genomic DNA Kit (DP304, TIANGEN, Beijing, China) according to the manufacturer’s instructions. DNA integrity was evaluated by 1% agarose gel electrophoresis, and DNA purity and concentration were measured on a NanoDrop 2000 (NanoDrop Technologies, Wilmington, DE, USA). Following Sangster and Luksenburg (2021), we verified the identity of our mitogenome sequence of *P.chinensis* with reference sequences of three commonly used markers in songbird systematics: NADH dehydrogenase subunit 2 (nad2, 1041 bp; n=1406, incl. 14 *P.chinensis*), part of cytochrome c oxidase subunit I (cox1, 696 bp; n=778, incl. two *P.chinensis*), and cytochrome b (cob, 1143 bp; n=939, incl. four *P.chinensis*). In each of these analyses, which were conducted with maximum likelihood analysis using a GTR+G+I model, our sequence of *P.chinensis* clustered with the reference sequences of *P.chinensis*, indicating that our sample was correctly identified.

### ﻿Genome sequencing, assembly and annotation

The *P.chinensis* DNA library was sequenced by Shanghai Personal Biotechnology Co., Ltd (Shanghai, China) using an Illumina NovaSeq with 300 bp paired-ends. The mitogenome was sequenced by next-generation sequencing. Raw sequence data were deposited into the GenBank database (https://www.ncbi.nlm.nih.gov/genbank/) with the accession number MT457820. Assembly of the mitochondrial genome was completed using A5-miseq version 2.0 (Coil et al. 2014). The tRNA genes were verified using the MITOS WebServer (Bernt et al. 2013) (http://mitos2.bioinf.unileipzig.de/index.py) and tRNAscan-SE 2.0 (Lowe and Chan 2016) using the default settings for the vertebrate mitochondrial genetic code. The tRNA secondary structures were predicted by tRNAscan-SE. Protein-coding regions were identified using the open reading frame (ORF) finder (Pombert et al. 2004) on the NCBI website with settings for the vertebrate mitochondrial genetic code and translated into putative proteins using GenBank. Base compositions were calculated and relative synonymous codon usage (RSCU) values were analyzed with MEGA v.7.0 (Kumar et al. 2016). Composition skew was calculated using the formula “AT-skew = (A–T) / (A+T)” and “GC-skew = (G–C) / (G+C)” (Perna and Kocher 1995). A graphical map of the mitogenome was drawn using the CGView Server (http://stothard.afns.ualberta.ca/cgview_server/index.html) (Stothard and Wishart 2005).

### ﻿Phylogenetic analyses

At present, only a small number of complete mitogenomes of species of the Leiothrichidae are available from GenBank. Therefore, the phylogenetic position of *P.chinensis* within Leiothrichidae was determined by comparing the 13 PCGs identified in *P.chinensis* to those of 13 other species of Leiothrichidae from six genera: *Pterorhinus*, *Ianthocincla*, *Garrulax*, *Trochalopteron*, *Liocichla* and *Leiothrix* (Table 1). *Alaudaarvensis* (GenBank Accession No. JQ322641) was used as an outgroup (Zhang et al. 2018a). Sequences of three species, *Pterorhinusalbogularis* (NC_037464), *Pterorhinuspoecilorhynchus* (NC_028082) and *Trochalopteronmilnei* (NC_041141), were excluded because each of these represented a chimera with DNA of two different species (Sangster and Luksenburg 2021). A sequence of “*Pterorhinusperspicillatus*” (NC_026068) was misidentified on GenBank and was renamed *Pterorhinuspectoralis*, based on Sangster and Luksenburg (2021).

**Table 1.** List of the 17 Leiothrichidae species and one outgroup used in this study with their GenBank accession numbers.

Family	Genus	Species	GenBank No.	References
Alaudidae	*Alauda*	*Alaudaarvensis* Linnaeus, 1758	JQ322641	Qian et al. 2013
Leiothrichidae	*Pterorhinus*	*Pterorhinussannio* Swinhoe, 1867	NC_028186	Unpublished
		*Pterorhinuscourtoisi* Ménégaux, 1923	NC_065197	Unpublished
		*Pterorhinusalbogularis* Gould, 1836	NC_037464	Unpublished
		*Pterorhinuslanceolatus* Verreaux J, 1871	KR818090	Qi et al. 2016a
		*Pterorhinuspoecilorhynchus* Gould, 1863	NC_028082	Qi et al. 2016b
		*Pterorhinuspectoralis* Gould, 1836	NC_026068	Unpublished
		*Pterorhinuschinensis* Scopoli, 1786	MT457820	**This study**
	*Ianthocincla*	*Ianthocinclamaxima* Verreaux, 1870	MZ129308	Unpublished
		*Ianthocinclaocellata* Vigors, 1831	NC_027657	Zhou et al. 2016a
		*Ianthocinclacineracea* Godwin-Austen, 1874	NC_024553	Xue et al. 2016
	*Garrulax*	*Garrulaxcanorus* Linnaeus, 1758	KT633399	Huang and Zeng 2016
	*Trochalopteron*	*Trochalopteronelliotii* Verreaux, 1870	NC_034373	Zhou et al. 2016b
		*Trochalopteronaffine* Blyth, 1843	NC_029402	Huang et al. 2016
		*Trochalopteronmilnei* David, 1874	NC_041141	Zhang et al. 2018b
	*Liocichla*	*Liocichlaomeiensis* Riley, 1926	KU886092	Unpublished
	*Leiothrix*	*Leiothrixlutea* Scopoli, 1786	MN356265	Unpublished
		*Leiothrixargentauris* Hodgson, 1837	HQ690245	Unpublished

The nucleotide sequences of the 13 PCGs of all 15 mitogenomes were concatenated and aligned using Clustal X in MEGA v.7.0 under the default parameters (Larkin et al. 2007). Phylogenetic analyses were performed by Bayesian inference (BI) and maximum likelihood (ML) methods. The Bayesian information criterion (BIC) in jModelTest v.0.1.1 was used to determine the optimal nucleotide substitution model, which was GTR+G+I (Santorum et al. 2014). The BI tree was produced using MrBayes v.3.2.1 with four Markov chains running simultaneously for 400,000 generations, sampling every 100 generations and discarding the first 25% as burn-in (Ronquist et al. 2012). The ML tree was produced using RAXMLGUI v.1.5b3 (Guindon and Gascuel 2003). A total of 1000 replicates were performed with the GTR+GAMMA substitution model. The resulting phylogenetic trees were visualized in FigTree v.1.2.2 (Rambaut and Drummond 2022).

## ﻿Results

### ﻿Mitogenome organization

**Figure 1.**
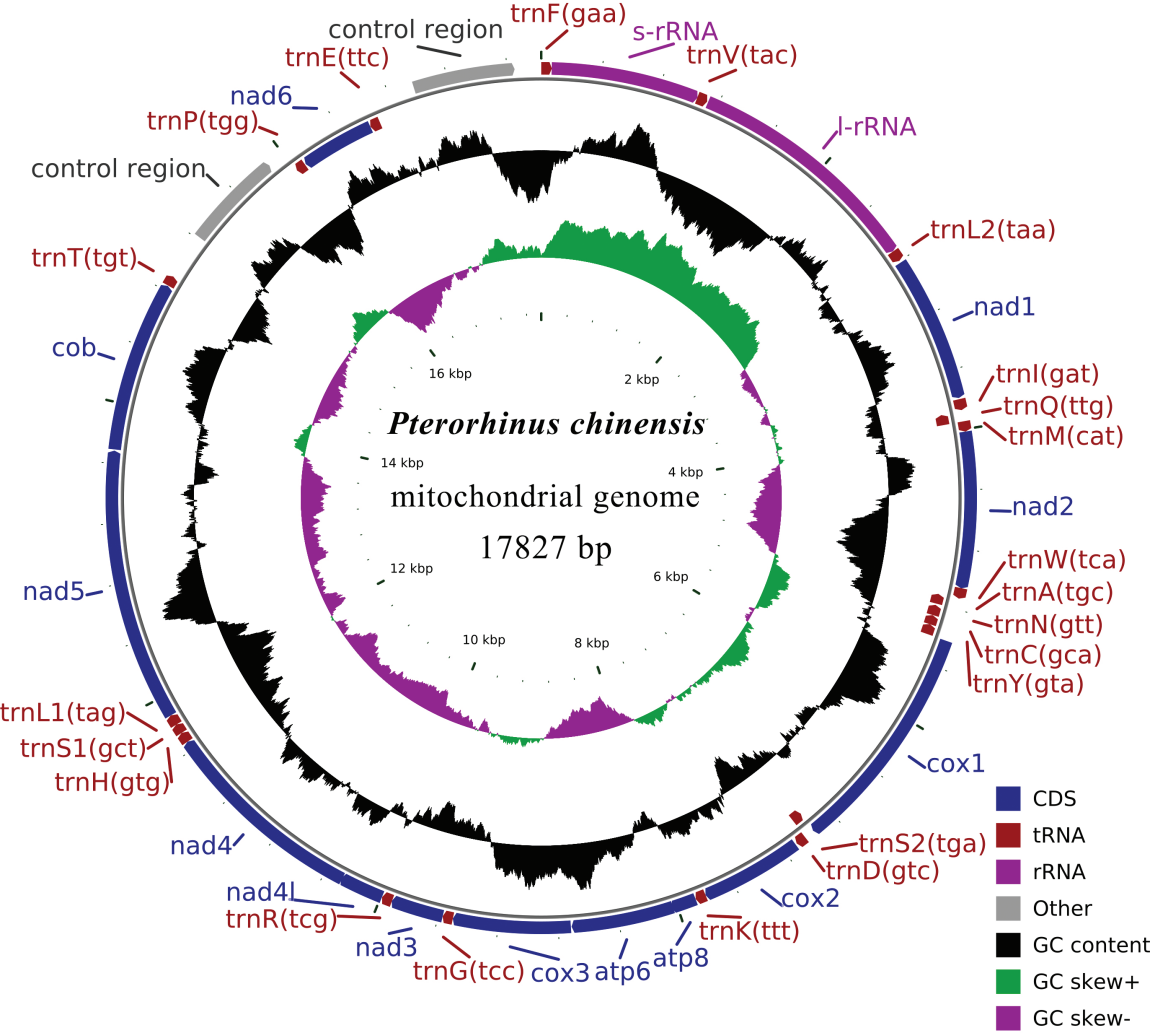
Gene map of the *P.chinensis* mitogenome.

The mitochondrial genome of *P.chinensis* is a typical closed-circular and double-stranded DNA molecule of 17,827 bp in length (Fig. 1). It contained the 37 typical mitochondrial genes (13 PCGs, 22 tRNAs and 2 rRNAs) and 2 control regions. Most gene sequences were on the H-strand including 12 PCGs, 14 tRNAs, 2 rRNAs and 2 control regions; however, nad6 and 8 tRNAs (trnA, trnC, trnE, trnN, trnP, trnQ, trnS2, and trnY) were encoded on the L-strand (Fig. 1, Table 2). The mitochondrial gene order of *P.chinensis* corresponded to nad5/cob/trnT/CR1/trnP/nad6/trnE/CR2/trnF/12S. Thus, there were two similar control regions, and the rearrangement type was “duplicate CR”. The mitogenome contained 33 overlapping nucleotides that were 1–10 bp in length and located in 10 pairs of neighboring genes. A comparison of all genes revealed the longest overlap (10 bp) between atp8 and atp6. A total of 978 intergenic nucleotides were found in 22 locations, ranging in size from 1 to 311 bp. The longest intergenic spacer (311 bp) was located between trnT and CR1.

**Table 2.** Annotation of the complete mitogenome of *P.chinensis*.

Gene	Strand	Start	Stop	Length (bp)	Intergenic length	Anticodon	Start codon	Stop codon
trnF	H	1	70	70	-1	GAA		
12S	H	70	1056	987	-1			
trnV	H	1056	1125	70	7	TAC		
16S	H	1133	2722	1590	1			
trnL2	H	2724	2798	75	14	TAA		
nad1	H	2813	3790	978	8		ATG	TAA
trnI	H	3799	3872	74	5	GAT		
trnQ	L	3878	3948	71	1	TTG		
trnM	H	3948	4016	69	0	CAT		
nad2	H	4017	5057	1041	-1		ATG	TAA
trnW	H	5057	5127	71	1	TCA		
trnA	L	5129	5197	69	10	TGC		
trnN	L	5208	5280	73	1	GTT		
trnC	L	5282	5347	66	-1	GCA		
trnY	L	5347	5417	71	1	GTA		
cox1	H	5419	6969	1551	-9		ATG	AGG
trnS2	L	6961	7033	73	4	TGA		
trnD	H	7038	7106	69	10	GTC		
cox2	H	7117	7800	684	0		ATG	TAA
trnK	H	7801	7870	70	1	TTT		
atp8	H	7872	8039	168	-10		ATG	TAA
atp6	H	8030	8713	684	5		ATG	TAA
cox3	H	8719	9502	784	0		ATG	T(AA)
trnG	H	9503	9571	69	0	TCC		
nad3	H	9572	9922	351	-1		ATG	TAA
trnR	H	9922	9991	70	1	TCG		
nad4l	H	9993	10289	297	-7		ATG	TAA
nad4	H	10283	11660	1378	0		ATG	T(AA)
trnH	H	11661	11730	70	0	GTG		
trnS1	H	11731	11796	66	-1	GCT		
trnL1	H	11796	11866	71	0	TAG		
nad5	H	11867	13684	1818	8		ATG	AGA
cob	H	13693	14835	1143	3		ATG	TAA
trnT	H	14839	14907	69	311	TGT		
CR1	H	15219	15882	664	126			
trnP	L	16009	16077	69	6	TGG		
nad6	L	16084	16602	519	1		ATG	TAG
trnE	L	16604	16675	72	297	TTC		
CR2	H	16973	17651	679	175			

The nucleotide composition of the complete mitogenome was as follows (Table 3): A = 29.45%, T = 23.26%, G = 14.48%, and C = 32.81%. The A + T content (52.71%) was substantially higher than that of G + C (47.29%). The overall AT-skew and GC-skew in the *P.chinensis* mitogenome were 0.12 and -0.39, respectively. The GC-skew, except for tRNAs, was slightly negative (-0.39 to -0.11), showing a higher occurrence of C than G. In contrast, the overall AT-skew, except for the 2 control regions, was slightly positive (0.01 to 0.24), showing a higher occurrence of A than T.

**Table 3.** Composition and skew values for *P.chinensis*.

Region	Size (bp)	A%	C%	T%	G%	A+T%	G+C%	AT-skew	GC-skew
mtDNA	17,827	29.45	32.81	23.26	14.48	52.71	47.29	0.12	-0.39
PCGs	11,369	27.47	33.73	24.13	14.67	51.60	48.40	0.06	-0.39
tRNAs	1546	28.96	20.71	28.18	22.15	57.14	42.89	0.01	0.03
rRNAs	2577	33.18	25.89	20.36	20.57	53.54	46.46	0.24	-0.11
CRs	1343	20.86	31.55	30.27	17.32	51.13	48.87	-0.18	-0.29

### ﻿Protein-coding genes

The total length of all PCGs in the mitogenome of *P.chinensis* was 11,369 bp and this accounted for 63.77% of the entire *P.chinensis* mitogenome. The A + T content in PCGs was 51.60% (Table 3). The gene with the highest number of base pairs was nad5 (1818 bp), and the gene with the lowest number was atp8 (168 bp). In addition, atp8 and atp6 shared 10 nucleotides; atp6 and cox3 had an interval of 5 nucleotides; nad4L and nad4 shared 7 nucleotides; and nad5 and cob had an interval of 8 nucleotides (Table 2). The initiation codon used for all PCGs was ATG for methionine. The predominantly used stop codons used were TAA (nad1, nad2, cox2, atp8, atp6, nad3, nad4l and cob). Whereas nad6 ended with TAG, cox1 ended with AGG, and nad5 ended with AGA. Incomplete stop codons (T**) were detected for cox3, and nad4 in *P.chinensis*.

**Figure 2.**
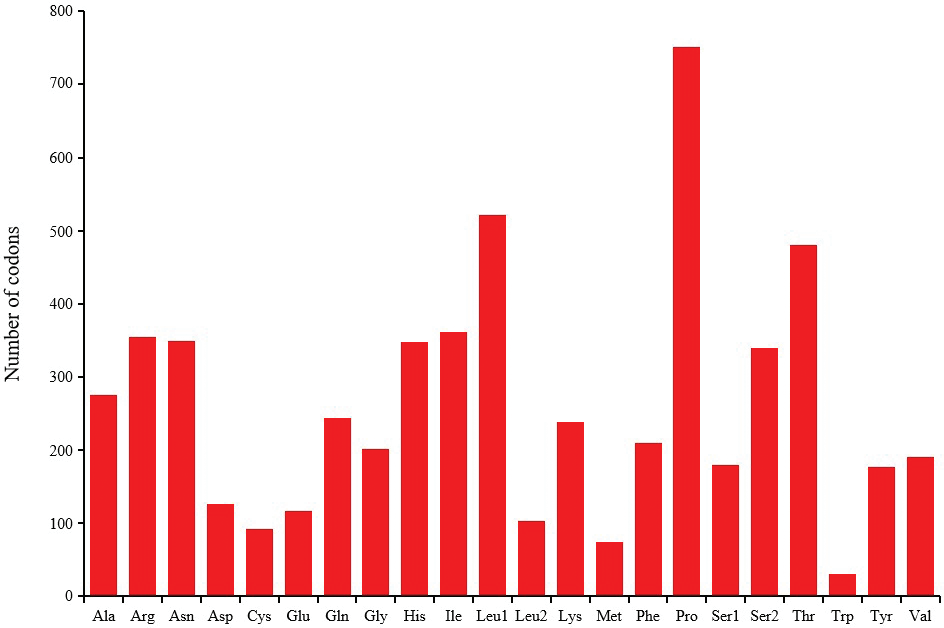
Codon distribution of the *P.chinensis* mitogenome. Numbers on the Y-axis refer to the total number of codons and codon families are provided on the X-axis.

**Figure 3.**
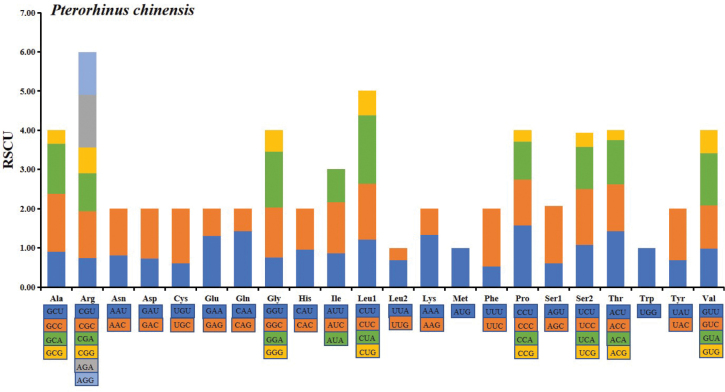
The relative synonymous codon usage (RSCU) of the *P.chinensis* mitogenome. Codons are shown on the X-axis and RSCU values are shown on the Y-axis.

RSCU values for the *P.chinensis* mitogenome for the third position are shown in Table 4. The total number of codons in PCGs was 5942. Codons encoding Trp were rare, while those encoding Pro were most frequent (Fig. 2). The codons Glu (GAA), Gln (CAA), and Lys (AAA) were mainly composed of T or A + T, while Gly (GGA), Leu1 (CUA) and Val (GUA) had a high G + C content (Fig. 3).

**Table 4.** Codon number and relative synonymous codon usage (RSCU) of *P.chinensis* mitochondrial protein-coding genes (PCGs).

Codon	Count	RSCU	Codon	Count	RSCU	Codon	Count	RSCU	Codon	Count	RSCU
UUU(F)	56	0.53	UCU(S)	93	1.08	UAU(Y)	61	0.69	UGU(C)	28	0.61
UUC(F)	154	1.47	UCC(S)	123	1.42	UAC(Y)	116	1.31	UGC(C)	64	1.39
UUA(L)	72	0.69	UCA(S)	93	1.08	UAA(*)	87	1.21	UGA(*)	78	1.08
UUG(L)	31	0.30	UCG(S)	31	0.36	UAG(*)	51	0.71	UGG(W)	30	1.00
CUU(L)	126	1.21	CCU(P)	281	1.57	CAU(H)	167	0.96	CGU(R)	44	0.74
CUC(L)	149	1.43	CCC(P)	209	1.17	CAC(H)	181	1.04	CGC(R)	71	1.20
CUA(L)	181	1.74	CCA(P)	173	0.97	CAA(Q)	173	1.42	CGA(R)	57	0.96
CUG(L)	65	0.63	CCG(P)	52	0.29	CAG(Q)	71	0.58	CGG(R)	39	0.66
AUU(I)	103	0.86	ACU(T)	172	1.43	AAU(N)	141	0.81	AGU(S)	53	0.61
AUC(I)	156	1.30	ACC(T)	143	1.19	AAC(N)	208	1.19	AGC(S)	126	1.46
AUA(I)	102	0.85	ACA(T)	136	1.13	AAA(K)	158	1.33	AGA(R)	80	1.35
AUG(M)	74	1.00	ACG(T)	30	0.25	AAG(K)	80	0.67	AGG(R)	64	1.08
GUU(V)	47	0.98	GCU(A)	62	0.90	GAU(D)	46	0.73	GGU(G)	38	0.76
GUC(V)	53	1.11	GCC(A)	102	1.48	GAC(D)	80	1.27	GGC(G)	64	1.27
GUA(V)	63	1.32	GCA(A)	87	1.27	GAA(E)	76	1.31	GGA(G)	72	1.43
GUG(V)	28	0.59	GCG(A)	24	0.35	GAG(E)	40	0.69	GGG(G)	27	0.54

Note: * represents stop codon.

### ﻿Ribosomal and transfer RNA genes

The small 12S rRNA was 987 bp, with an A + T content of 50.80%; whereas the large 16S rRNA of *P.chinensis* was 1590 bp in length, with an A + T content of 54.40%. The two rRNAs were located between trnF and the trnL2, and isolated by trnV (Fig. 1 and Table 2).

**Figure 4.**
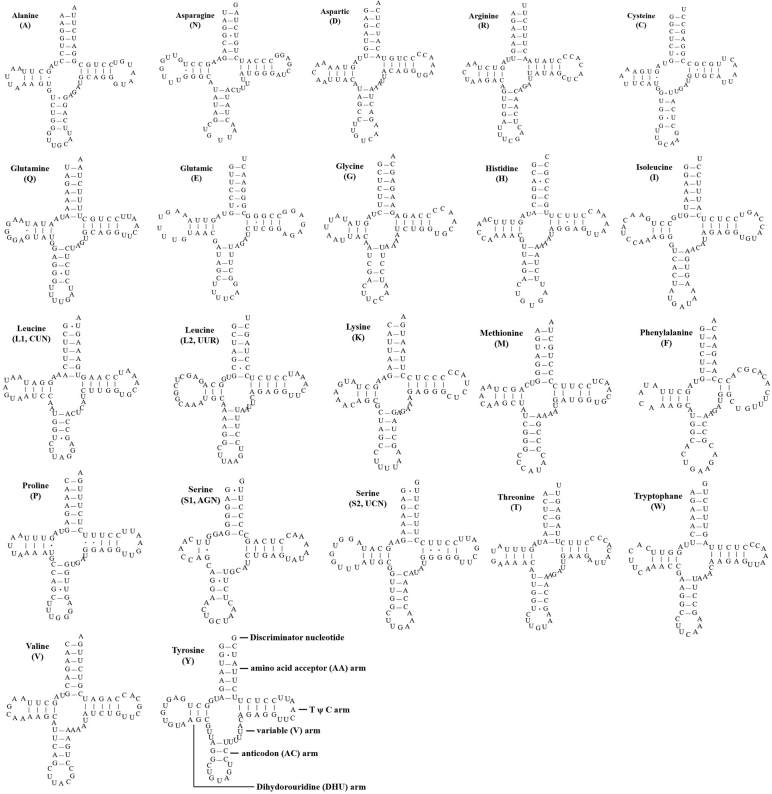
Secondary structures of the 22 transfer RNA genes of *P.chinensis*.

The total length of tRNA ranges for *P.chinensis* was 1546 bp, and this accounted for 8.70% of the total mitogenome. The average length of tRNA was 70 bp, the longest was trnL2 (75 bp), and the shortest was trnC and trnS1 (66 bp) (Table 2). All tRNAs in the mitogenome of *P.chinensis* had the canonical cloverleaf structure with slight variation in sequence length of the stem regions of the main arms. The A + T content of the 22 tRNAs was 57.14%. The mismatched base pairs G-U, A-C, U-U, A-A, and C-C were found in all *P.chinensis* tRNAs except for trnR, trnI, trnK, trnF, trnW, and trnV (Fig. 4).

### ﻿Non-coding sequencing

The mitogenome of *P.chinensis* contained two similar control regions (CR1, CR2), which were 664bp and 679bp in length, respectively. They were located between trnT and trnF genes, separated by trnP, nad6, and trnE genes (Fig. 1). The base composition of CR1 was 20.93% A, 30.72% T, 17.32% G, and 31.02% C. The A + T content (51.65%) was higher than the G + C content (48.34%). The base composition of CR2 was 20.76% A, 30.49% T, 17.08% G, and 31.66% C. The A + T content (51.25%) was higher than the G + C content (48.74%).

### ﻿Phylogenetic analyses

**Figure 5.**
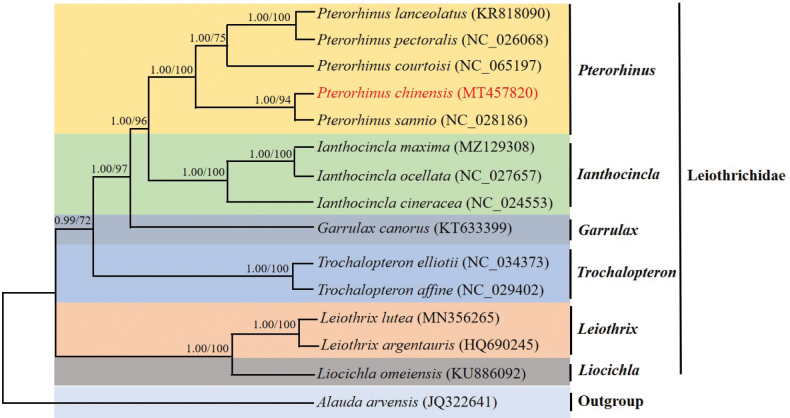
Phylogenetic relationships of Leiothrichidae species determined using concatenated nucleotide sequences of 13 PCGs. Both BI and ML analyses produced identical tree topologies. Values at nodes are BI posterior probabilities and ML bootstrap values, respectively.

Phylogenetic analysis based on concatenated alignments of 13 PCGs of 15 species was carried out. The topology of the phylogenies reconstructed by BI and ML analyses was identical (Fig. 5). To gain insight into the phylogenetic interrelationships within Leiothrichidae, we obtained the concatenated nucleotide sequences of 13 PCGs from 14 species of Leiothrichidae, including five *Pterorhinus*, three *Ianthocincla*, one *Garrulax*, two *Trochalopteron*, two *Leiothrix* and one *Liocichla*.

Within Leiothrichidae, we found that the phylogenetic relationships among the six genera were: *Leiothrix* and *Liocichla* were sister to the other four genera. *Garrulax* and the clade (*Pterorhinus* + *Ianthocincla*) formed a clade (posterior probability 1.0, 97% bootstrap support), and *Leiothrix* and *Liocichla* were sister taxa (posterior probability 1.0, 100% bootstrap support).

In addition, *P.sannio* was sister to *P.chinensis* (posterior probability 1.0, 94% bootstrap support). The (*P.chinensis* + *P.sannio*) and ((*P.lanceolatus* + *P.pectoralis*) + *P.courtoisi*) formed a well-supported clade (posterior probability 1.0, 100% bootstrap support). Meanwhile, our results showed that *P.chinensis* was distantly related to *Garrulax*.

## ﻿Discussion

### ﻿Mitogenome characteristics

In this study, the complete mitochondrial genome of *P.chinensis* was characterized for the first time. As in other species of Leiothrichidae, the mitogenome of *P.chinensis* consisted of 13 PCGs, two rRNAs, 22 tRNAs, and two control regions (Qian et al. 2013; Huan et al. 2016). Except for eight tRNAs (trnA, trnC, trnE, trnN, trnP, trnQ, trnS2 and trnY) and the nad6 gene, all the genes were coded on the H-strand, similar to that in most other vertebrates (Huan et al. 2016). Our study documents gene rearrangement in *P.chinensis*. Gibb et al. (2007) proposed the following nomenclature for avian mitochondrial gene orders: (1) Ancestral gene order; (2) remnant CR2 gene order; (3) duplicate CR gene order; and (4) duplicate tRNA^Thr^-CR gene order. The mitogenome of *P.chinensis* includes two similar control regions and therefore we suggest that this represents a duplicate CR gene order. As in some other Passeriformes species, the two control regions (CR1 and CR2) were positioned between the trnT and trnF genes, and were separated by trnP, nad6, and trnE, with length of 664 and 679 bp, respectively (Huan et al. 2016). There were also intergenic spacers and overlaps between genes, as is seen in other birds (Dong et al. 2018).

The A + T content in the complete mitochondrial genome of *P.chinensis* was 52.71%, in line with the typical base bias of vertebrates (Huang and Zeng 2016). All PCGs of the mitogenome of *P.chinensis* were initiated with ATG, similar to those of most Passeriformes species (Huan et al. 2016). However, they were terminated with five types of stop codons, including TAA (nad1, nad2, cox2, atp8, atp6, nad3, nad4l, and cob), AGG (cox1), AGA (nad5), TAG (nad6), and incomplete stop codons T** (cox3 and nad4), as in as other species of Leiothrichidae (Qian et al. 2013; Huan et al. 2016). For the incomplete stop codons, the missing nucleotides may be the result of post-transcriptional polyadenylation, which is common in animal mitogenomes and could produce functional stop codons by polycistronic transcription cleavages and polyadenylation mechanisms (Ojala et al. 1981). The anticodons of all tRNAs in the mitogenome of *P.chinensis* were identical to those observed in most vertebrates (Sun et al. 2020). Furthermore, mismatched base pairs were identified in the stems of 22 different tRNAs, most of which are G-U pairs, which can form a weak bond in tRNAs and non-canonical pairs in tRNA secondary structures (Gutell et al. 2002). rRNAs includes 12S rRNA and 16S rRNA, with lengths of 987 bp and 1590 bp, respectively. As in most vertebrates, these *rRNA* genes were located between trnF and trnL2, and isolated by trnV (Lu et al. 2013).

### ﻿Phylogenetic analyses

Mitochondrial sequences are widely used to infer phylogenetic relationships among vertebrate species (Anderson et al. 1981; Miya et al. 2001), including birds (e.g., Jønsson et al. 2019). In this study, we explored the phylogenetic relationship among members of Leiothrichidae based on 13 PCGs. Our study supports the phylogenetic relationships of ((((*Pterorhinus* + *Ianthocincla*) + *Garrulax*) + *Trochalopteron*) + (*Leiothrix* + *Liocichla*)). These results partially agree with the topologies inferred by Cibois et al. (2018) and Cai et al. (2019). The latter two agree with our findings that (1) *Garrulax* is sister to (*Pterorhinus* + *Ianthocincla*) and that (2) *Leiothrix* and *Liocichla* are more closely related to each other than either is to *Garrulax*, *Pterorhinus* or *Ianthocincla*. However, our results differ from those of Cibois et al. (2018) and Cai et al. (2019) in placing *Trochalopteron* closer to *Garrulax*, *Pterorhinus* or *Ianthocincla* than to *Leiothrix* and *Liocichla*, whereas Cibois et al. (2018) and Cai et al. (2019) inferred a closer relationship of *Trochalopteron* to *Leiothrix* and *Liocichla* than to *Garrulax*, *Pterorhinus* or *Ianthocincla*.

Our analyses support the reallocation of *P.chinensis* from the genus *Garrulax* to *Pterorhinus*, consistent with previous studies (Cibois et al. 2018; Cai et al. 2019). Currently, published mitochondrial genome data of species of Leiothrichidae are scarce. In order to better understand the phylogenetic relationships among Leiothrichidae, additional sequences of mitochondrial genomes are warranted.

## ﻿Additional information

### Data availability

All of the data that support the findings of this study are available in the main text.
